# *Rhizobiales* as functional and endosymbiontic members in the lichen symbiosis of *Lobaria pulmonaria* L.

**DOI:** 10.3389/fmicb.2015.00053

**Published:** 2015-02-10

**Authors:** Armin Erlacher, Tomislav Cernava, Massimiliano Cardinale, Jung Soh, Christoph W. Sensen, Martin Grube, Gabriele Berg

**Affiliations:** ^1^Institute of Environmental Biotechnology, Graz University of TechnologyGraz, Austria; ^2^Institute of Plant Sciences, University of GrazGraz, Austria; ^3^Department of Biochemistry and Molecular Biology, University of CalgaryCalgary, AB, Canada; ^4^Institute of Molecular Biotechnology, AG Computational Biotechnology, Graz University of TechnologyGraz, Austria

**Keywords:** *Rhizobiales*, lichen symbiosis, *Lobaria pulmonaria*, metagenomics, *Rhizobiales*-specific FISH probe, endosymbiont

## Abstract

*Rhizobiales* (*Alphaproteobacteria)* are well-known beneficial partners in plant-microbe interactions. Less is known about the occurrence and function of *Rhizobiales* in the lichen symbiosis, although it has previously been shown that *Alphaproteobacteria* are the dominating group in growing lichen thalli. We have analyzed the taxonomic structure and assigned functions to *Rhizobiales* within a metagenomic dataset of the lung lichen *Lobaria pulmonaria* L. One third (32.2%) of the overall bacteria belong to the *Rhizobiales*, in particular to the families *Methylobacteriaceae, Bradyrhizobiaceae*, and *Rhizobiaceae*. About 20% of our metagenomic assignments could not be placed in any of the *Rhizobiales* lineages, which indicates a yet undescribed bacterial diversity. SEED-based functional analysis focused on *Rhizobiales* and revealed functions supporting the symbiosis, including auxin and vitamin production, nitrogen fixation and stress protection. We also have used a specifically developed probe to localize *Rhizobiales* by confocal laser scanning microscopy after fluorescence *in situ* hybridization (FISH-CLSM). Bacteria preferentially colonized fungal surfaces, but there is clear evidence that members of the *Rhizobiales* are able to intrude at varying depths into the interhyphal gelatinous matrix of the upper lichen cortical layer and that at least occasionally some bacteria also are capable to colonize the interior of the fungal hyphae. Interestingly, the gradual development of an endosymbiotic bacterial life was found for lichen- as well as for fungal- and plant-associated bacteria. The new tools to study *Rhizobiales*, FISH microscopy and comparative metagenomics, suggest a similar beneficial role for lichens than for plants and will help to better understand the *Rhizobiales*-host interaction and their biotechnological potential.

## Introduction

Lichen symbioses are estimated to cover up to 8% of the global land surface. Many habitats colonized by lichens are characterized by unfavorable environmental conditions, such as low nutrient availability and/or high temperature fluctuations (Ahmadjian, 1995). Although lichens are able to resist extreme environmental conditions via a dormant stage, they are highly specialized for their habitats and vulnerable to slight changes in the microclimate (or air pollution), which can easily disrupt the integrity of the fine-tuned symbiotic interplay. Lichen symbioses appear as composite organisms with a shape-forming fungus (the mycobiont) and a photosynthetic partner (the photobiont), which is often sheltered by complex fungal structures, into which a complex, stable and thallus-specific microbiome is incorporated. Recently, a bacterial microbiome was identified as a third component of this symbiosis (Grube et al., 2009). Lichens are densely colonized by diverse and host-specific communities of bacteria that occur in specific ecological niches of their hosts (Cardinale et al., 2008, 2012a,b; Grube et al., 2009). Specific above-ground niches in higher plants comprize for example the phyllo- and rhizosphere, or the endosphere (Ryan et al., 2008; Berg et al., 2014). However, lichens do not produce the same organs as found in plants, which develop their organs from meristems. Lichens instead produce a thallus of densely conglutinated fungal hyphae which can form foliose, filamentous, crustose, leprose, squamulose, gelatinous, or fruticose shapes (Grube and Hawksworth, 2007), each hosting specific sets of ecologically specific niches, which can be occupied by bacteria.

*Rhizobiales* are well-studied associates of plants; they commonly exert beneficial functions for their hosts by providing various nutrients, phytohormons as well as precursors for essential plant metabolites (Ivanova et al., 2000; Delmotte et al., 2009; Verginer et al., 2010). The order contains many genera of nitrogen-fixing, methanotrophic, legume-nodulating, microsymbiotic bacteria (Jourand et al., 2004; Garrity et al., 2005). Recently, nitrogen-fixation was shown not to be limited to *Rhizobiales* in leguminous plants, but also to be expressed within various endophytic compartments of non-leguminous plants (Fischer et al., 2012). Besides their almost ubiquitous presence with higher plants, *Rhizobiales* are also found associated with mosses and lichens (Lundberg et al., 2012; Vorholt, 2012; Erlacher et al., in press). Pink-pigmented-facultative-methylotrophs (PPFMs) are a specific group of *Rhizobiales*, which can affect the host metabolism including production of vitamins and phytohormones, such as auxines and cytokinines (Ivanova et al., 2000; Delmotte et al., 2009). *Methylobacterium* spp. can utilize methanol emitted by the plants, methylamine and further C2, C3, and C4 compounds as solely carbon and energy source (Green and Bousfield, 1983; Lidstrom and Chistoserdova, 2002). Schauer and Kutschera (2013) suggest that ferns, liverworts and moss protonemata have an intimate association with methylobacteria, and they argue that the haploid phases of cryptogames are preferred host organisms of these pink-pigmented microbial phytosymbionts. However, less is known for lichen-associated *Rhizobiales*, including methylobacteria. We postulate that they also play a beneficial role in the lichen symbiosis.

According to recent publications, *Rhizobiales* are a particularly common order on lichens (Bates et al., 2012; Cardinale et al., 2012b). Although there are several reports about endofungal bacteria in ascomycetous fungi (Bertaux et al., 2005; Sharma et al., 2008), until know there is no evidence for them in lichens (Grube and Berg, 2009). While *Alphaproteobacteria* have been detected by fluorescence *in situ* hybridization and confocal laser scanning microscopy in lichens (Cardinale et al., 2008; Grube et al., 2009), there are no suitable FISH probes available to specifically stain the order *Rhizobiales*, except RHIZ1244 (according to Probebase) (http://www.microbial-ecology.net/probebase; accession nr. pB-02665; Thayanukul et al., 2010). *In silico* analysis using probematch (https://rdp.cme.msu.edu/probematch/) revealed that the RHIZ1244 probe detection spectrum is incomplete and fails to recognize important families such as *Methylobacteriaceae, Bradyrhizobiaceae*, or *Beijerinckiaceae*.

**Figure 1:**
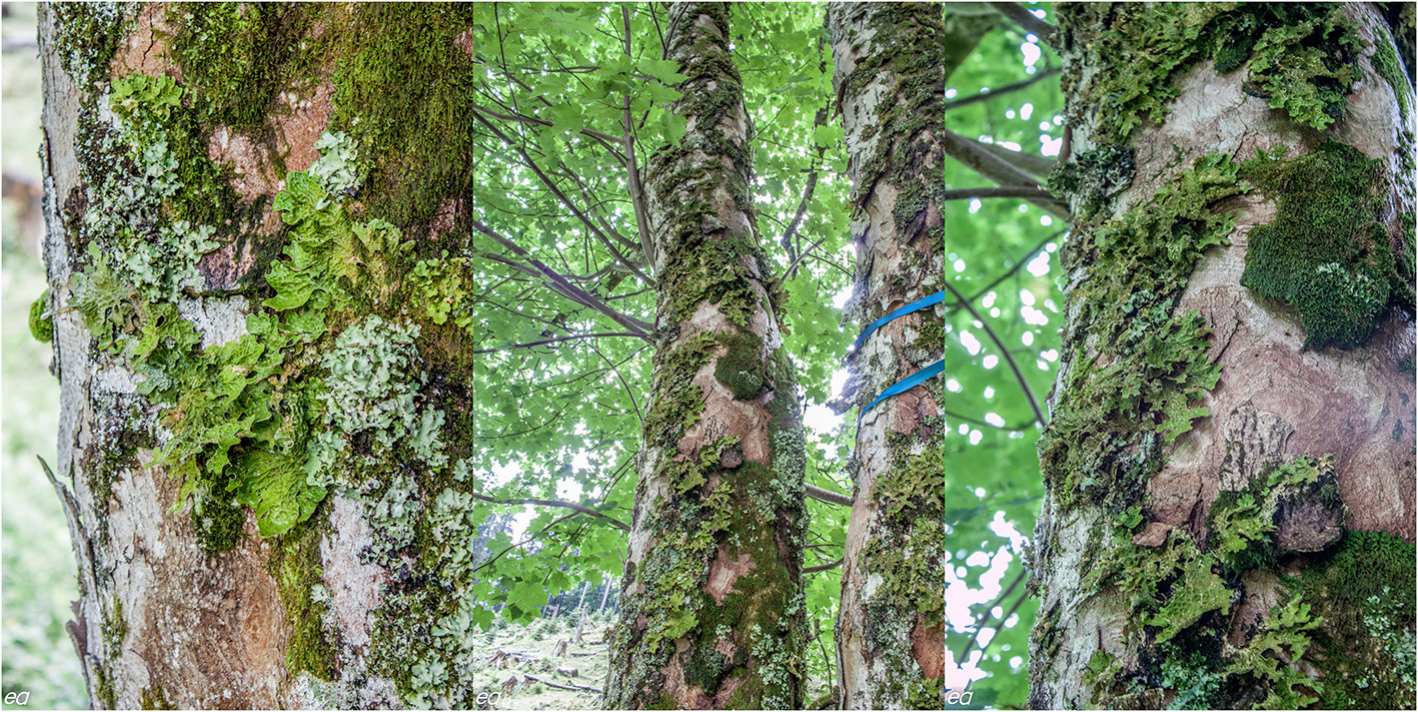
***Lobaria pulmonaria* from a mountain maple in an Abieti-Fagetum forest in Johnsbach (Austria) at an altitude of 1200 m above sea level**. (Styria, Austria, 47°32′29.7″N 14°37′36.6″E).

The lung lichen, *Lobaria pulmonaria* L., is a tripartite lichen, with one ascomycete fungus hosting both a dominant green algal partner (*Dictyochloropsis reticulata*) and a minor cyanobacterial partner (internal herds of *Nostoc*). This lichen is known as a sensitive biological indicator of air pollution that experienced a massive decline in Europe during the twentieth century (Scheidegger and Goward, 2002). Nonetheless, it may develop prolific populations in suitable cool and humid habitats, both by the efficient spread with symbiotic propagules and by its growth rate, which is one of the highest among all lichens (Figure 1). In this work, we have investigated *L. pulmonaria* collected in the high montane forest zone in Alps. We pursued a metagenomic approach to assess functional diversity of *Rhizobiales* associated with this lichen species and used *in situ* visualization to localize and reveal colonization strategies of this bacterial order. For this purpose, we designed a novel FISH probe to efficiently and specifically target members of *Rhizobiales*.

## Materials and methods

### Sampling

*L. pulmonaria* samples were collected in two mountain forests from mountain maple (*Acer pseudoplatanus* L.) in Austria (Styria, Gstatterboden, 47°34′20″N, 14°35′4.4″E and Styria, Johnsbach, 47°32′29.7″N 14°37′36.6″E). Ten individual lichen thalli were collected and stored in sterile plastic bags on ice.

### *Lobaria pulmonaria* metagenome and analyzes

All metagenome-based analyzes were carried out on the assembled dataset described in a previous study by Grube et al. (2015). The number of actual contigs used for the *in silico* FISH-probe evaluation was 368,424, while 28,526 contigs assigned to the taxon *Rhizobiales* were used for the functional analysis of *Rhizobiales*. CLUSTER CONTROL (Stocker et al., 2004) was used to search with the blastn algorithm for FISH-probe matches within the dataset (*e*-value cutoff = 1.6). The assembled metagenomic dataset is publicly available on MG-RAST (http://metagenomics.anl.gov; project ID: 4529136.3). To obtain taxonomic assignments, the Tera-BLASTN program (www.timelogic.com/documents/TeraBLAST_2009.pdf) was run on the 368,424 contigs, using TimeLogic (Active Motif, Carlsbad, CA, USA) DeCypher boards against the “nt” (nucleotide sequence) database from NCBI (ftp://ftp.ncbi.nlm.nih.gov/blast/db). The blastn results were imported into MEGAN (Metagenome Analyzer, v4.70.4) (Huson et al., 2011) to produce several taxonomy profiles. For functional analysis, we used a similar approach as above but used Tera-BLASTX (www.timelogic.com/documents/TeraBLAST_2009.pdf), which was run against the “nr” (non-redundant protein sequence) database from NCBI (ftp://ftp.ncbi.nlm.nih.gov/blast/db). The blastx results were imported into MEGAN (v4.70.4) as well for functional analysis. The 28,526 contigs that were previously assigned to *Rhizobiales* were used for assignment of SEED functions (Overbeek et al., 2005) within MEGAN. SEED-based analysis allows hierarchical organization of complete and partial gene sequences allocated within the utilized contig collection and thus quantification of specific functions on different levels.

### *In silico* analysis of rRNA-targeted oligonucleotide probes

ProbeBase (http://www.microbial-ecology.net/probebase; Loy et al., 2007) was used to screen for available FISH probes targeting the order *Rhizobiales*. RDP Probe Match (https://rdp.cme.msu.edu/probematch; Cole et al., 2005) and the Silva RNA database using TestProbe 3.0 (http://www.arb-silva.de/search/testprobe; Quast et al., 2013) with taxonomy browser were utilized to evaluate the amplitude and coverage of available and designed FISH probes. The Probe sequences (5′–3′) were aligned (reversed and complement search allowed) to RDP and Silva SSU r119 databases with the REFNR sequence collection.

### Fluorescence *in situ* hybridization combined with confocal laser scanning microscopy (FISH-CLSM)

FISH-CLSM was applied on *L. pulmonaria* samples to investigate colonization patterns of *Rhizobiales* and all bacteria. Within 3 h after collection, samples were fixed with 4% paraformaldehyde and 1x phosphate-buffered saline (PBS) (3:1 ratio, respectively) for 6 h at 4°C. Fixed *Lobaria* thallus samples were cut with a cryotome.

We designed FISH probe RHIZ3r (Table 1) specific to our metagenomic data. The oligonucleotide sequence based on the Primer 3r (Nishio et al., 1997) was synthetized and labeled with a Cy5 fluorochrome (Biomers, Wiener Neudorf, Austria). FISH was applied according to Cardinale et al. (2008) to visualize and decipher the nature of the correlations detected by metagenomics. Briefly, the cryosections were transferred into 1.5 ml Eppendorf tubes and rinsed with 1x PBS. Lysozyme (1 mg/ml; Sigma-Aldrich, St. Louis, MO, USA) treatment was applied and incubated at RT for 10 min. After an ethanolic series (50–70–96% EtOH solutions; 3 min each) samples were rinsed and further washed for 3 min with ice-cold 1x PBS. All hybridizations were performed at 43°C for 2 h in a buffer containing 0.9 M NaCl, 0.02 M Tris-HCl, 0.01% sodium dodecyl sulfate, 10–50% (Table 1) ultrapure formamide (FA; Invitrogen), and 5.0 ng of each FISH probe μl^−l^ (pH 8). An equimolar mixture of Cy3-labeled EUB338, EUB338-II and EUB338-III probes (Amann et al., 1990; Daims et al., 1999) was used for staining all Bacteria. RHIZ3r and RHIZ1244 (Thayanukul et al., 2010) was used to stain taxa within the bacterial order *Rhizobiales*. NONEUB probes (Wallner et al., 1993) labeled to fluorochromes analogous to the positive probes were used as negative controls. The hybridization buffer was replaced by a prewarmed (44°C) washing buffer [20 mM Tris-HCl, 450/46/18 mM NaCl (10/40/50% FA), and 5 mM EDTA (for 40% and 50% FA)] and incubated for 15 min in a water bath (44°C). The hybridization and washing step were repeated sequentially for the utilized FISH-probes in dependency of the specific FA requirements. After eliminating the washing buffer the sections were again rinsed with ice-cold double-distilled H_2_O in order to remove residual salt crystals. FISH stained samples were transferred on optical slides, dried and mounted with SlowFade Gold antifadent (Molecular Probes, Eugene, USA). For visualization a Leica TCS SPE confocal laser-scanning microscope (Leica Microsystems, Mannheim, Germany) was used. Autofluorescence and additional calcofluor white (Sigma-Aldrich) staining of the lichen tissues was used for imaging the host structures. The fluorescent dyes Cy3, and Cy5 labeling the FISH probes were sequentially excited with 532 and 635 nm laser beams. Autofluorescence and calcofluor staining was excited with a 405 nm laser beam. The confocal stacks were acquired with a LeicaACS APO 40x oil CS objective lens (NA, 1.15) and a Leica ACS APO 63x oil CS objective lens (NA, 1.30) and for each field of view, an appropriate number of optical slices were acquired within a Z-step ranging from 0.15 to 0.5 μm. The software Imaris 7.3 (Bitplane, Zurich, Switzerland) was used for imaging and post-processing of the confocal stacks and maximum projections. Adobe Photoshop (Adobe Systems Inc., USA) was used to label the final figures.

**Table 1:** **Oligonucleotide probes utilized for FISH in this study**.

**Name**	**Sequence (5′-3′)**	**Fluorochrome**	**Target**	**Formamide (% at 43°C)**	**References**
EUB338^*^	GCTGCCTCCCGTAGGAGT	Cy3	Most bacteria	10	Amann et al., 1990
EUB338II^*^	GCAGCCACCCGTAGGTGT	Cy3	*Planctomycetales*	10	Daims et al., 1999
EUB338III^*^	GCTGCCACCCGTAGGTGT	Cy3	*Verrucomicrobiales*	10	Daims et al., 1999
NONEUB^**^	ACTCCTACGGGAGGCAGC	Cy5 or Cy3	/	^**^	Wallner et al., 1993
RHIZ3r	GGCTTATCACCGGCAGTCTCC	Cy5	*Rhizobiales*	40	Nishio et al., 1997
RHIZ1244	TCGCTGCCCACTGTCACC	Cy5	*Rhizobiales*	50	Thayanukul et al., 2010

* *Probes were used in equimolar concentration*.

** *NONEUB was applied as negative control; formamide concentrations were analog to the positive FISH probes*.

## Results

### Analysis of *lobaria*-associated *rhizobiales*

**Figure 2:**
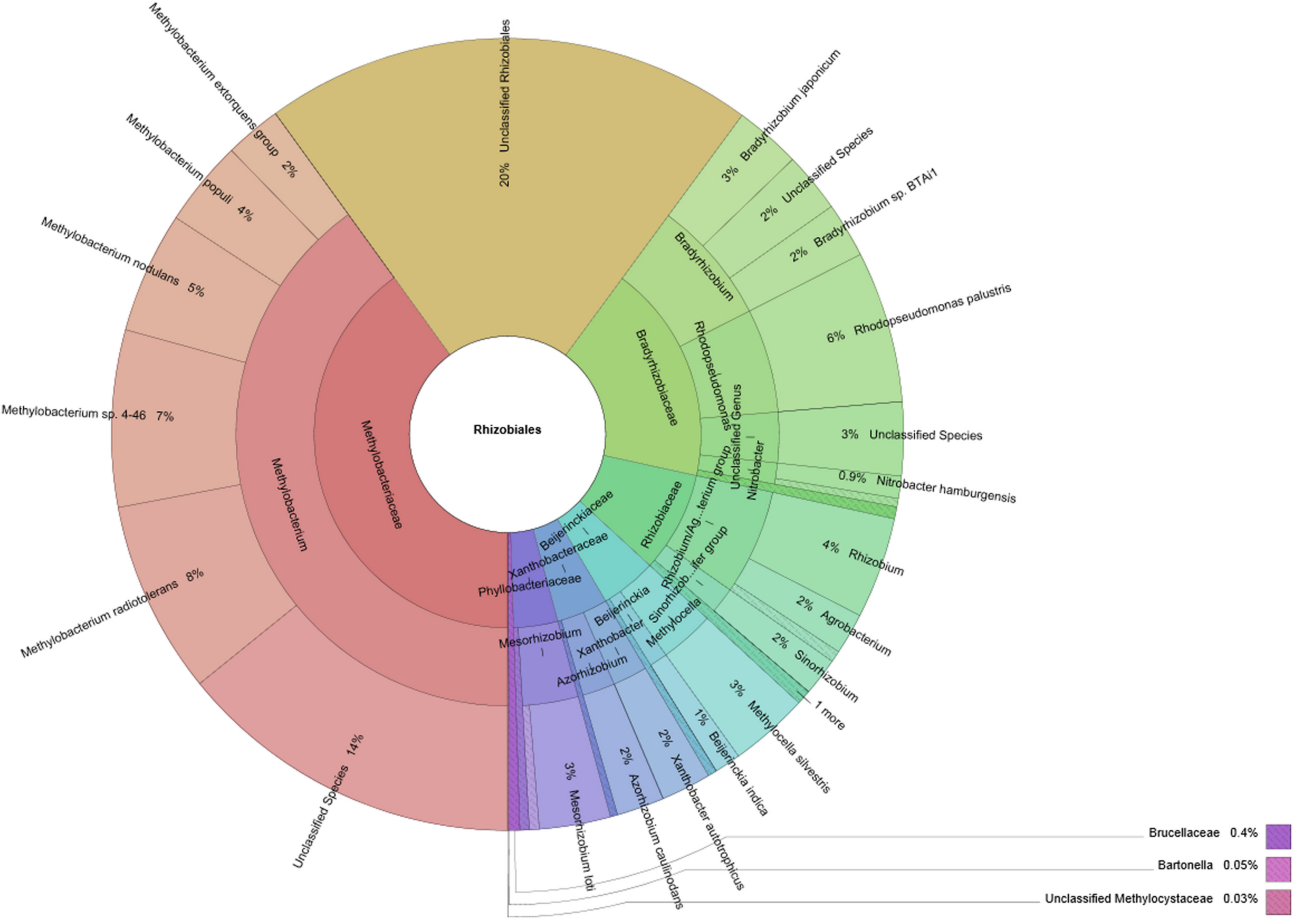
***Rhizobiales* taxa within the *Lobaria*-associated metagenome are presented in a multi-level chart**. Taxonomic information was retrieved through blastn assignments of assembled contigs, followed by processing with MEGAN. Circles represent taxonomic classifications in ascending order up to the species level (outermost circle). Less abundant taxa are listed outside the charts together with their relative abundance.

An assembled *Lobaria*-associated metagenome consisting of 362,424 contigs was utilized for taxonomic and functional studies of assigned *Rhizobiales*. In total, 88,602 contigs were assigned to bacteria. *Alphaproteobacteria* was the most frequently identified bacterial phylum in the metagenome and comprized 53,688 contigs (46.8% of all identified cellular organisms and 60.6% of identified bacteria). Of these, 28,526 assigned contigs belong to the predominant *Rhizobiales* (32.2% of identified bacteria), with families *Methylobacteriaceae* (11,421 contigs or 12.9% of identified bacteria), *Bradyrhizobiaceae* (5230 contigs or 5.9% of identified bacteria), and *Rhizobiaceae* (2403 contigs or 2.7% of identified bacteria). *Methylobacterium* was the only identified genus of *Methylobacteriaceae* and *Methylobacterium radiotolerans* (8% of all *Rhizobiales*) the most frequent species. Less frequent species were identified as *M. nodulans, M. populi* and members of the *M. extorquens* group. A total of 21% of present *Rhizobiales* was assigned to the cluster *Methylobacterium* sp. 4–46 or remained unclassified. Identified genera within the family of *Bradyrhizobiaceae* were more diverse and represented by four distinctive genera: *Bradyrhizobium* (7% of all *Rhizobiales*), *Rhodopseudomonas* (6% of all *Rhizobiales*), *Nitrobacter* (1% of all *Rhizobiales*) and *Oligotropha* (0.5% of all *Rhizobiales*). Three percent of all *Rhizobiales* remained unclassified genera of the *Bradyrhizobiaceae* family. The most abundant species within *Bradyrhizobiaceae* was identified as *Rhodopseudomonas palustris* (6% of all *Rhizobiales*). *Rhizobiaceae* included the *Rhizobium/Agrobacterium* group (6% of all *Rhizobiales*) and the *Sinorhizobium/Ensifer* group (2% of all *Rhizobiales*). Less abundant *Rhizobiales* families were assigned to the genera of *Beijerinckiaceae* (5% of all *Rhizobiales*), *Xanthobacteraceae* (4% of all *Rhizobiales*), *Phyllobacteriaceae* (4% of all *Rhizobiales*) and *Brucellaceae* (0.4% of all *Rhizobiales*). A detailed taxonomic composition of *Rhizobiales* up to species level was visualized with Krona ((Ondov et al., 2011); Figure 2).

**Figure 3:**
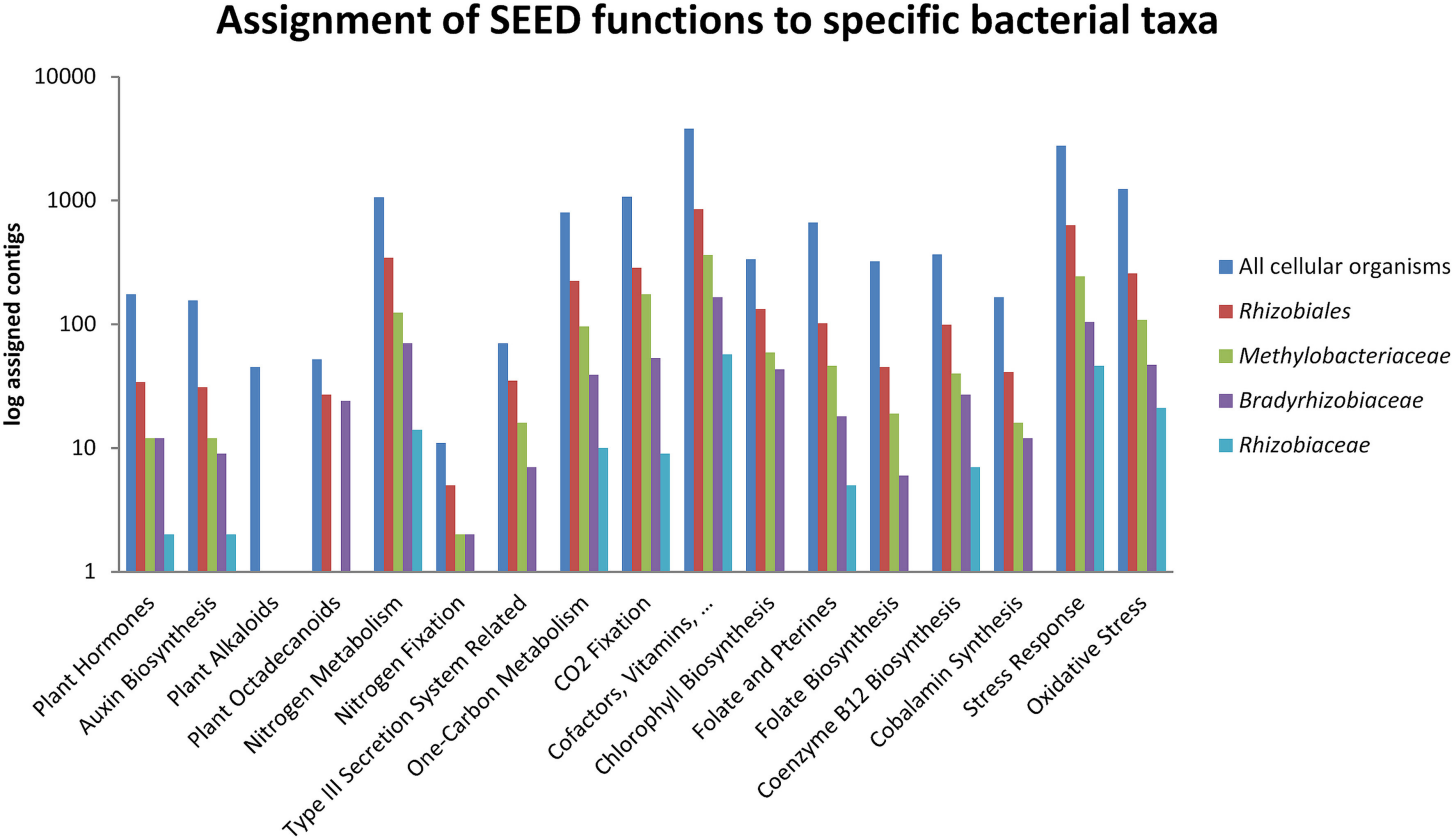
**Selected functions within the *Lobaria*-associated metagenome were assigned to taxa at order (*Rhizobiales*) and family (*Methylobacteriaceae, Bradyrhizobiaceae* and *Rhizobiaceae*) level together with hits without assignment to a specific taxon**. Taxonomic information was retrieved through blastx assignments of assembled contigs, followed by processing with MEGAN.

SEED-based functional analysis (Figure 3) focused on *Rhizobiales* and its three most abundant families. Included clustering and hierarchical organization of identified functions was utilized to retrieve quantitative information for highly abundant taxa. The abundance of function-related genes assigned to specified taxonomic ranks was compared to their overall occurrence in the entire metagenome. Thereby we obtained a comprehensive overview of functions with high relevance to the symbiotic system (Table S1). *Lobaria*-associated *Rhizobiales* were shown to be involved in all candidate functions (except biosynthesis of plant alkaloids). They were found to be involved in the biosynthesis of auxins and plant octadecanoids. Notably *Bradyrhizobiaceae* accounted for 24 contigs assigned to biosynthesis of plant octadecanoids, while *Methylobacteriaceae* only accounted for one contig containing this function. Nitrogen fixation was represented by 5 contigs assigned to *Rhizobiales*, and one assigned to either *Bradyrhizobiaceae* or *Methylobacteriaceae*. Type III secretion systems were found in 35 contigs, with 16 assigned to *Methylobacteriaceae*, 7 assigned to *Bradyrhizobiaceae*, one assigned to *Rhizobiaceae* and 11 without assignment to a specific family. One-carbon metabolism and carbon dioxide fixation were represented within *Rhizobiales* by 224 and 286 contigs, respectively. Biosynthesis of cofactors, vitamins, prosthetic groups and pigments was particularly frequent and represented by 848 contigs within *Rhizobiales*. Notably *Bradyrhizobiaceae* were found to contribute to chlorophyll biosynthesis (59 contigs), synthesis of folate and pterines (46 contigs) and coenzyme B12 biosynthesis (99 contigs). Conversely, *Rhizobiaceae* were rather underrepresented with 5 contigs assigned to synthesis of folate and pterines and 7 contigs assigned to coenzyme B12 biosynthesis. Overall stress response was found within 632 contigs associated with *Rhizobiales*. *Methylobacteriaceae* accounted for 243 contigs, while *Bradyrhizobiaceae* and *Rhizobiaceae* accounted for 104 and 46 contigs, respectively. Response to oxidative stress was present with 257 contigs (108 assigned to *Methylobacteriaceae*, 47 to *Bradyrhizobiaceae* and 21 to *Rhizobiaceae*).

### Evaluation of the RHIZ3r FISH probe for *rhizobiales* staining

**Figure 4:**
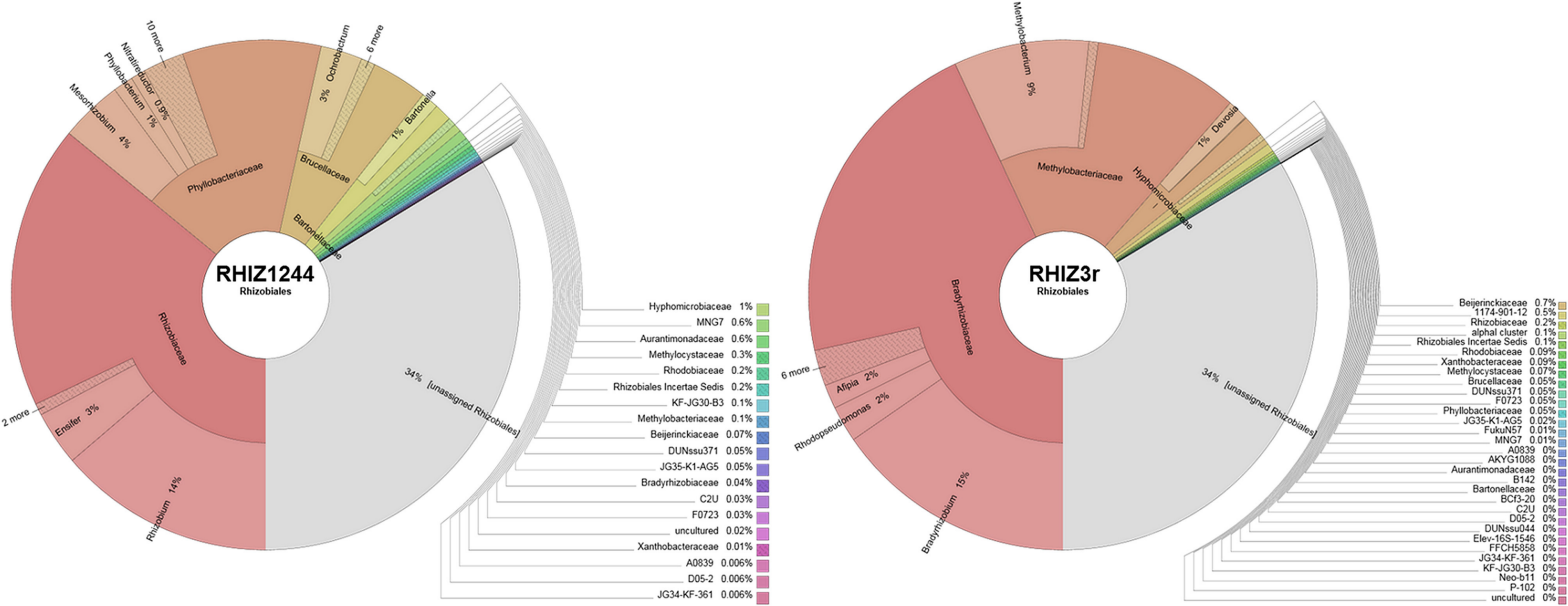
**TestProbe 3.0 (Silva ribosomal RNA database) analysis showing the targeted coverage of bacterial taxa in a comparison of RHIZ3r- and RHIZ1244- FISH Probes**. The maximum amount of mismatches was set to 0 (see also Table S3 for RDP Probmatch comparison).

Alignments to sequences of the Silva (Quast et al., 2013) and Probematch databases (Cole et al., 2005) revealed that the only available FISH probe RHIZ1244 targeting *Rhizobiales* was not suitable to label taxa retrieved in the *Lobaria* microbiome (Figure 4; Table S2). The designed FISH probe RHIZ3r, based on the primer 3r (Nishio et al., 1997), was therefore evaluated and we could demonstrate a high coverage for specific taxa, including the most abundant families *Methylobacteriaceae* and *Bradyrhizobiaceae* (Figure 4). According to the Probematch analysis (Table S3), RHIZ3r (11166 hits) shows slightly reduced coverage in the order *Rhizobiales* compared to RHIZ1244 (14312 hits). However, the latter probe does not match well with the highly abundant families *Bradyrhizobiaceae* (7/11453) and *Methylobacteriaceae* (5/9098 hits), whereas RHIZ3r performs much better in this respect (*Methylobacteriaceae*: 3821/9098 hits; *Bradyrhizobiaceae:* 5586/11453 hits; Table S3). *In silico* alignment of the sequence to genus level shows that the FISH probe RHIZ3r targets bacteria belonging to the order *Rhizobiales*, families *Methylobacteriaceae* (genus *Methylobacterium*, coverage: 86%) and *Bradyrhizobiaceae* (genus *Bradyrhizobium*, coverage: 99%; genus *Afipia*, coverage: 100%; genus *Nitrobacter*, coverage: 100%; genus *Oligotropha*, coverage: 100%; genus *Rhodoblastus*, coverage: 100%; genus *Rhodopseudomonas*, coverage: 92%). The results of the *in silico* analysis were confirmed by identification of excised SSCP bands amplified with primer RHIZ3r (Erlacher et al., in press) and with the metagenomic data.

### *in silico* evaluation of potential FISH probes for *rhizobiales* in the metagenome

We used Blastn to search for FISH probe binding sites within the entire assembled *Lobaria*-associated metagenome, and found different *Rhizobiales* taxa, including members of the families *Rhizobiaceae, Beijerinckiaceae, Bradyrhizobiaceae, Methylobacteriaceae, Methylocystaceae*, and *Phyllobacteriaceae*. Hits for non-targeted taxa included mostly unspecific chloroplast and plastid DNA as well as two hits for *Xanthomonadaceae* and one hit for *Pseudomonadaceae* (Table S2).

### Vizualisation of *lobaria*-associated *rhizobiales*

**Figure 5:**
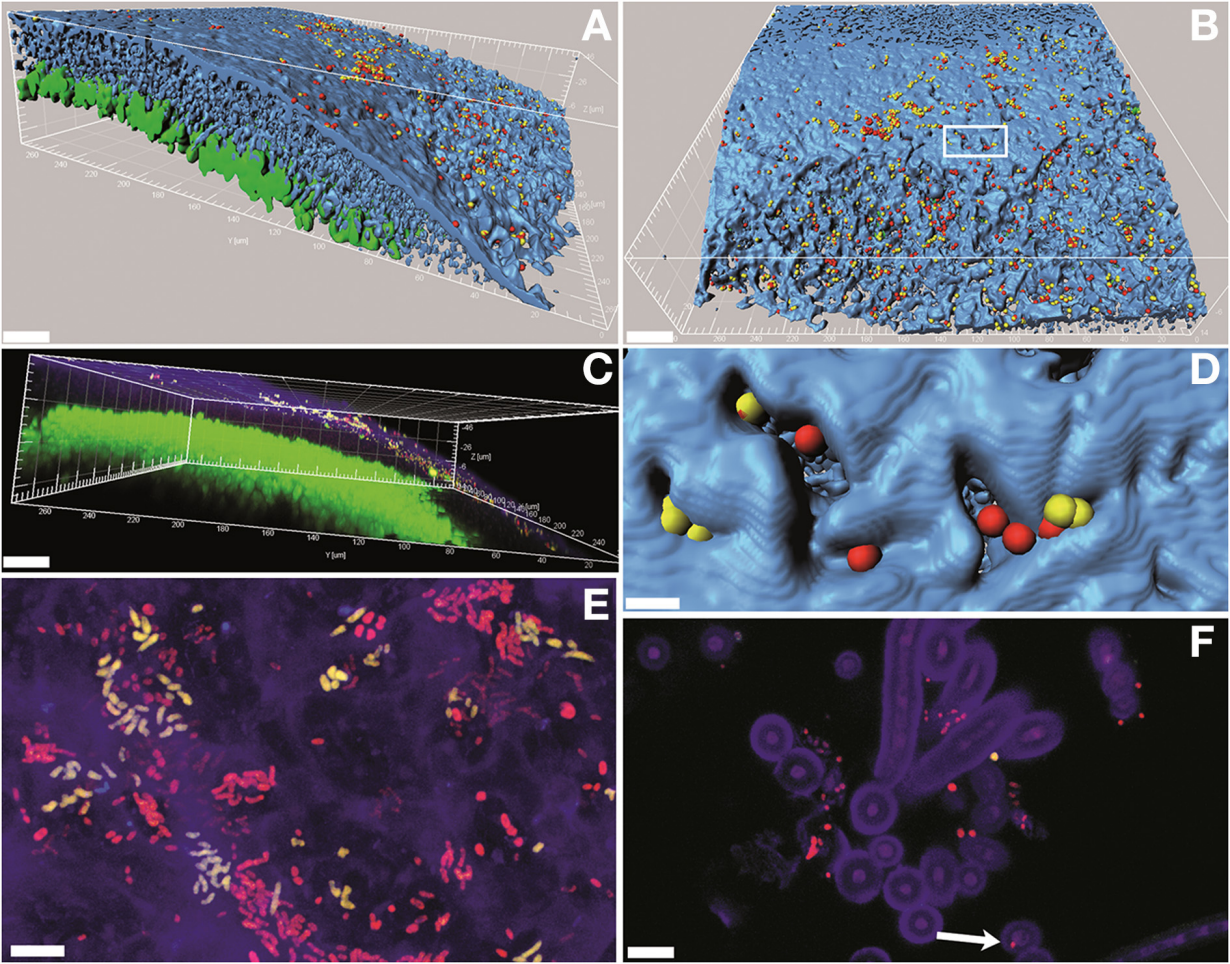
**Confocal laser scanning microscopy (CLSM) micrographs showing the bacterial colonization of the lichen *L. pulmonaria* stained by fluorescence *in situ* hybridization (FISH)**. Green, algae; blue/purple, fungi; yellow, *Rhizobiales*; red, other bacteria. (**A,B,D)**, three-dimensional models made of isosurfaces and spheres; **(C)**, volume rendering; **(E)**, maximum projection; **(F)**, single optical slice. In **(B)**: white box indicates the region shown in **(D)**. Arrow in **(F)** indicates an endophytic bacterial cell (see also Figure S1). Scale bars: **(A-C)** (30 μm); **(D-F)** (5 μm).

Fluorescence *in situ* hybridization with both the RHIZ3r and the EUB338-MIX probes resulted in unambiguously strong signals. Image analysis and three-dimensional reconstructions of confocal stacks showed that most of the bacteria colonize *L. pulmonaria* at the outer surface of the lichen cortex (Figures 5A–C). Mixed colonies formed by putative *Rhizobiales* and other bacteria were frequently detected (Figures 5A–C,E), and morphological diversity of bacteria was apparent (Figure 5E). The autofluorescent fungi and algae in *Lobaria* allowed us to reconstruct the host structure (Figures 5A,B,D). Close co-existence between the bacteria and the hydrophilic fungal cortex were observed (Figure 5D), whereas intra-thalline hydrophobic spaces as well as photobionts were not colonized by bacteria (Figures 5A,C). Free hyphae protruding from the lower surfaces were often covered by bacteria. In rare cases, bacteria colonized the hyphae internally (Figure 5F; Figures S1, S2).

## Discussion

Our new study provides a first insight into the functional potential of *Rhizobiales*, which are the predominant order of bacteria associated with the lichen symbiosis. *Rhizobiales* are responsible for more than one third of all bacterial taxonomic assignments. About 20% of our metagenomic assignments could not be placed in any of the *Rhizobiales* lineages, which indicates that there might be numerous yet undescribed bacterial diversity colonizing lichens. One taxonomically undescribed phylogenetic lineage of *Rhizobiales*, not present in our dataset, was detected in diverse lichens from North America, and named LAR1 (Hodkinson and Lutzoni, 2009). Most of the classified bacteria in our dataset belong to the families *Methylobacteriaceae, Bradyrhizobiaceae* and *Rhizobiaceae*, which are therefore expected to play an important role within the lichen symbiosis.

Because *Rhizobiales* are common in growing lichen parts, we argue that they could play a role in development and growth of lichens. This hypothesis is well supported by the potential functions encoded in the metagenomic contigs of *Alphaproteobacteria* and *Rhizobiales* in our dataset. SEED-based functional analysis revealed functions supporting the symbiosis, including auxin and vitamin production, nitrogen fixation and stress protection. Taxonomical assignments showed high proportions of beneficial nitrogen fixing at species level. However, we think that nitrogen-fixation is not a required rhizobial function in the *L. pulmonaria* symbiosis, because fixed nitrogen is provided by the associated cyanobacterial partners (which is located in clustered colonies, in so-called internal cephalodia), and because excessive nitrogen (e.g., agricultural contamination) is rather a problem affecting the survival of *L. pulmonaria* in many localities. It is therefore interesting to observe a significant number of contigs that is assigned to nitrogen metabolism. Metabolism related to cofactors and vitamin production is also well represented in our dataset, suggesting that the corresponding products are valuable to support the growing lichen thallus. In addition, the high abundance of *Methylobacterium* species might be a promising source to find novel compounds or bioconversion as in higher plants (Verginer et al., 2010). In comparison with a study of *Methylobacterium* spp. on mosses by Erlacher et al. (in press), using fingerprinting methods, we detected higher species diversity in the *L. pulmonaria* microbiome, which is also confirmed by the metagenomic data. Stress protection for the symbiosis by bacteria was detected, which seem to play a unique and important function of host-associated microbiomes. Stress protection was already detected for mosses (Bragina et al., 2014) but also for plant-associated bacteria (Alavi et al., 2013). The biotechnological potential of stress-protecting bacteria was already shown (Alavi et al., 2013; Berg et al., 2013), which shows new solutions for agriculture in a changing climate.

So far, colonization of lichens was mostly shown on surfaces of lichens (e.g., Cardinale et al., 2008). The present study clearly shows that *Rhizobiales* members are not restricted to the thallus surface. It is thus tempting to consider endobiotic life style of bacteria, similar to endophytism in plants. However, there are marked differences to an endophytic lifestyle of higher plants. Plants are typically characterized by a protective cuticula which forms a clear boundary between the plant and the external environment. By the cuticula internal tissues, plants are protected against uncontrolled water loss or contamination from external water, dirt, and from invasion of microorganisms. Such a layer is missing in thalline organisms such as mosses or lichen thalli (which are also known as “lower plants” or “cryptogams”). Both mosses and lichens belong to poikilohydric organisms, desiccating with atmospheric drought. Without a cuticula it is also more difficult to differentiate between endosphere and phyllosphere. The present data confirm that there is no clear external border of the lichen surface. We have already observed a depletion of bacterial abundance in other lichens but no qualitative differences when we analyzed bacterial associates of lichens after increasing duration of surface sterilization (unpublished data). By studying lichens we uncover interesting new insights about the endophytic strategies. In some lichens, the internal parts of lichens can be colonized. This is clearly shown in *Cladonia*, where hollow thalli are internally colonized by biofilm like bacterial communities (Cardinale et al., 2008). In cases of crust-forming lichens we observed that bacteria can partly enter the lateral parts of neighboring thallus segments (areoles; e.g., *Lecanora polytropa*, Grube et al., 2009). The case of *Lobaria* now shows that the external polysaccharide matrix between the hyphae of the lichens can, at varying depths, be penetrated by *Rhizobiales*. The loose aggregation of hyphae and the lack of a cuticula found in higher plants facilitate mutualistic bacterial colonization which gradually develops from ecto- to endo-symbiotic lifestyles. We have not observed bacteria so far in the algal layer or in the aerated medulla part beneath the algal layer. We suppose this is due to the fact that particularly the medulla layer of lichens has strongly hydrophobic surfaces (due to a hydrophobin cell layer, which enwrap the cells of the eukaryote partners). However, we also found first indications of endohyphal occurrences of bacteria in *L*. *pulmonaria* (Figure 5F; Figures S1, S2). While our findings of intracellular colonization of lichenized-fungal hyphae still require additional methodological prove to be validated, the endohyphal bacterial occurrence in non-lichenized fungi has repeatedly been found in very different lineages (e.g., Bertaux et al., 2005; Partida-Martinez and Hertweck, 2005; Sharma et al., 2008). Recent work sheds light on their diverse functions (Ghignone et al., 2012), and also revealed new details regarding how bacteria penetrate fungal hyphae (Moebius et al., 2014). We argue that occasional endohyphal bacteria in lichens might be particularly efficient strains to digest the rather thick fungal cell walls in lichens. This observation may also spur new interest in lichens as a bioresource for biotechnological applications.

Beneficial plant-microbe interactions were extensively studied in the past and reviewed by Berg (2009). Such interactions include diverse and important functions including the suppression of pathogens and the increase in plant growth and fitness. While the traits involved in bacterial adaption and exchange of particular metabolites to higher plants are partially deciphered (Vorholt, 2012), less is known about microbe-lichen interactions. Functional assignments from the metagenome suggest *Rhizobiales* as a vital component supporting the lichen symbiosis. Results indicate that they are able to supply auxiliary as well as essential metabolites to their host. This study is the first to relate the abundance of bacteria with potential functions of their representatives within the lichen structure. Our present study also provides first indications for lichen endosymbiosis.

## Conflict of interest statement

The authors declare that the research was conducted in the absence of any commercial or financial relationships that could be construed as a potential conflict of interest.
