# Supplementary material for: Rhizobiales as functional and endosymbiontic members in the lichen symbiosis of Lobaria pulmonaria L
Source: Front Microbiol. 2015 Feb 10;6:53. doi: 10.3389/fmicb.2015.00053 (PMC4322706; doi:10.3389/fmicb.2015.00053)

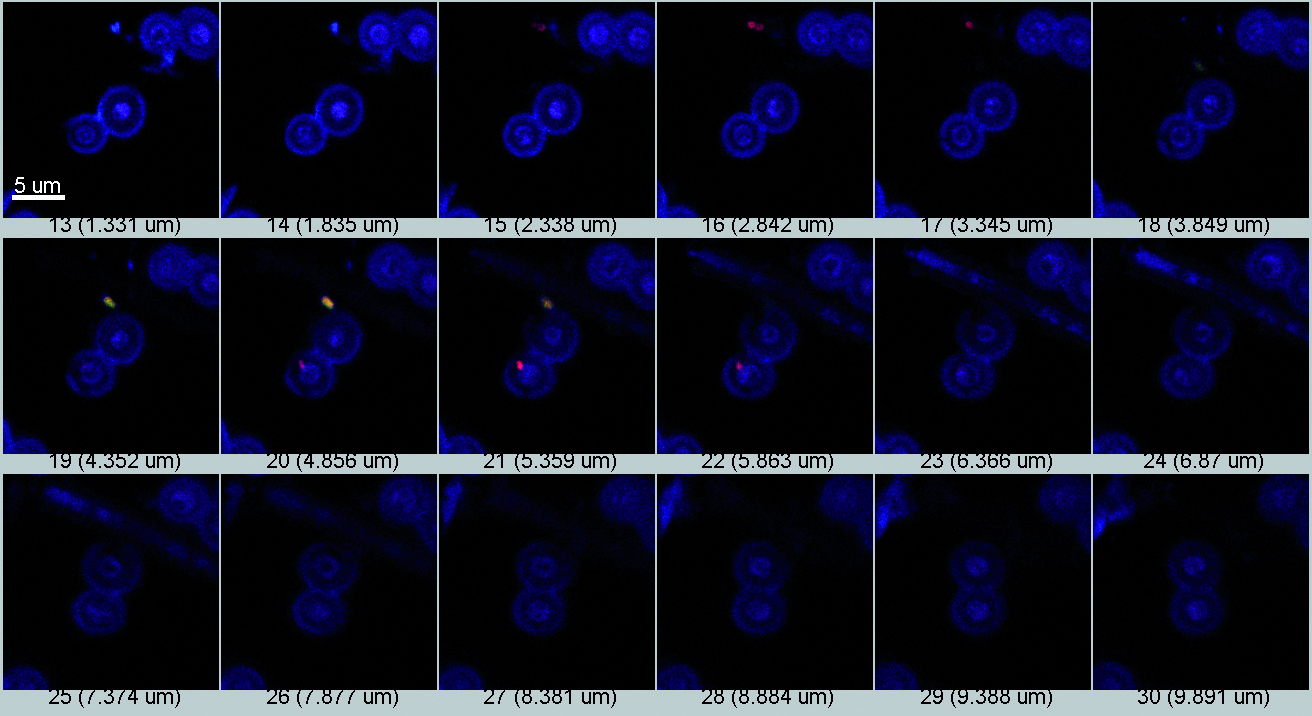


Figure S1 Endohyphal bacterial occurrence in *Lobaria pulomonaria*. Confocal microscopy image series showing a FISH-stained bacterial cell endophytically colonizing a fungal hypha. The distance between the confocal planes is 0.5 µm. Blue/purple = fungi (autofluorescence); yellow = RHIZ3r-FISH+EUB338-MIX stained bacteria; red = only EUB338-MIX stained bacteria.


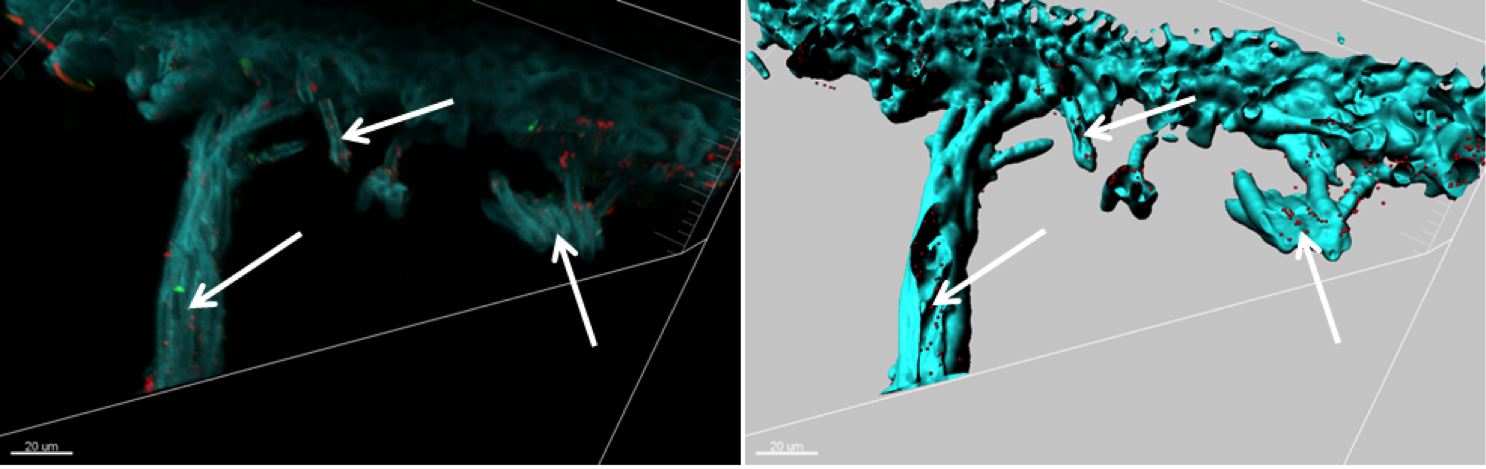


**B**

**A**

**D**

**C**

Figure S2 Endohyphal bacterial occurrence in *Lobaria pulomonaria*. Confocal microscopy volume (A) and iso-surface model (B) showing FISH-stained bacterial cells (red, arrows) endophytically colonizing fungal hyphae. The distance between the confocal planes is 0.5 µm.

Table S1 Assignment of SEED functions to bacterial taxa within the *Lobaria* metagenome. Functions were assigned to overall *Rhizobiales* and the three most abundant families therein. Higher SEED level functions are highlighted in grey with more specific functions thereunder (not highlighted).

Table S2 *In silico* evaluation of the FISH probe with the whole *Lobaria*-associated metagenome and blastn analysis.

Table S3 RDP Probematch analysis showing the comparison of RHIZ3r- and RHIZ1244- FISH Probes. Bold letters indicate taxa occurring in the *Lobaria* Metagenome; Asterisks indicate the two most dominant groups *Methylobacteriaceae* and *Bradyrhizobiaceae*. The Maximum amount of mismatches was set to 0.


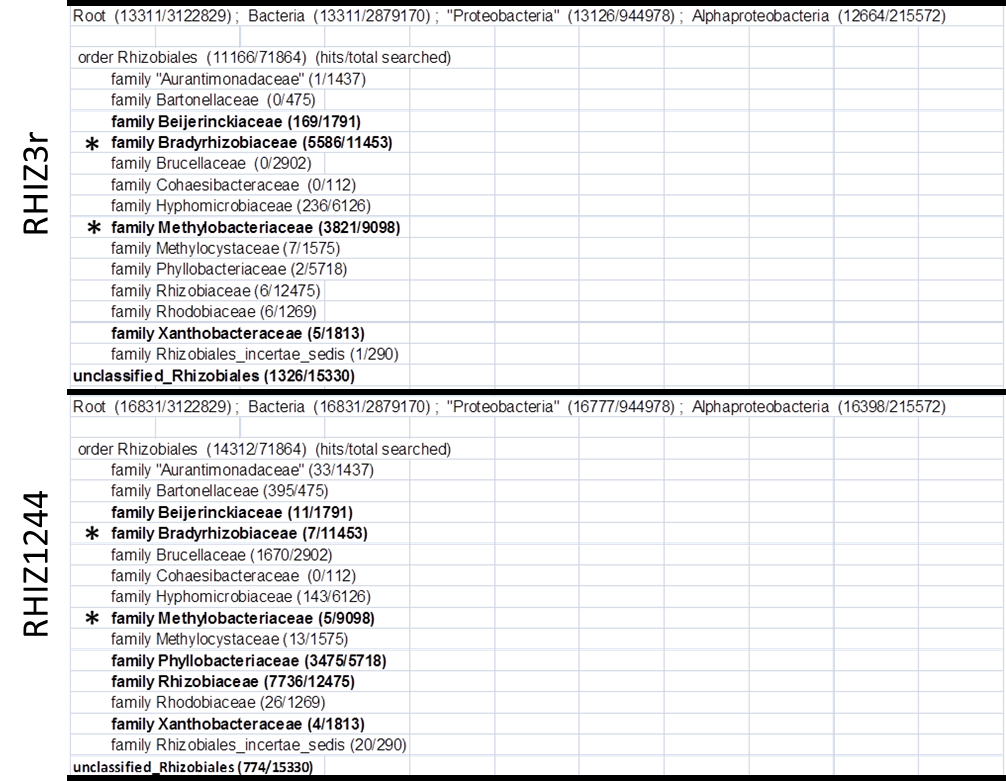

Supplement: Supplementary file 1 [file DataSheet1.DOCX]
